# A global perspective on stepping down chronic spontaneous urticaria treatment: Results of the Urticaria Centers of Reference and Excellence SDown‐CSU study

**DOI:** 10.1002/clt2.12343

**Published:** 2024-02-14

**Authors:** Murat Türk, Emek Kocatürk, Ragıp Ertaş, Luis Felipe Ensina, Silvia Mariel Ferrucci, Clive Grattan, Christian Vestergaard, Torsten Zuberbier, Marcus Maurer, Ana Maria Giménez‐Arnau

**Affiliations:** ^1^ Department of Chest Diseases Division of Allergy and Clinical Immunology Erciyes University School of Medicine Kayseri Turkey; ^2^ Clinic of Allergy and Clinical Immunology Kayseri City Education and Research Hospital Kayseri Turkey; ^3^ Institute of Allergology Charité – Universitätsmedizin Berlin Corporate Member of Freie Universität Berlin and Humboldt‐Universität zu Berlin Berlin Germany; ^4^ Fraunhofer Institute for Translational Medicine and Pharmacology ITMP Allergology and Immunology Berlin Germany; ^5^ Department of Dermatology Chronic Skin Diseases Unit University of Health Sciences Kayseri City Education and Research Hospital Kayseri Turkey; ^6^ Division of Allergy, Immunology, Rheumatology Federal University of São Paulo Sao Paulo Brazil; ^7^ Dermatology Unit Fondazione IRCCS Ca’ Granda Ospedale Maggiore Policlinico Milan Italy; ^8^ St John's Institute of Dermatology Guy's Hospital London UK; ^9^ Department of Dermatology Aarhus University Hospital Denmark Aarhus Denmark; ^10^ Department of Dermatology Hospital del Mar & Research Institute Barcelona Spain; ^11^ Universitat Pompeu Fabra Barcelona Spain

**Keywords:** antihistamine, chronic urticaria, cyclosporine, guideline, omalizumab, step down, urticaria control test

## Abstract

**Background:**

Although there have been significant advances in the treatment of chronic spontaneous urticaria (CSU) in recent years, there remains a lack of clear guidance on when and how to step down treatment in responders. This study aims to investigate stepping down approaches of different steps of CSU treatment from a global perspective.

**Methods:**

“Stepping down chronic spontaneous urticaria treatment” (SDown‐CSU) is an international, multicenter, observational, cross‐sectional, survey‐based study of the Urticaria Centers of Reference and Excellence (UCARE) network. The questionnaire included 48 questions completed by physicians in the UCARE network.

**Results:**

Surveys completed by 103 physicians from 81 UCAREs and 34 countries were analyzed. Seventy‐eight percent of the participants responded that they had a national urticaria management guideline written by their professional societies and 28% responded that they had to operate under a regulatory guideline proposed by central health funding organizations. Seventy‐two and 58.7% of these national recommendations do not contain any detailed information on when and/or how CSU treatment should be discontinued. There was a lack of detailed information on antihistamines and cyclosporine in particular. A predefined maximum duration was generally not applicable to omalizumab and cyclosporine (81% and 82%, respectively). Nearly all UCAREs step down omalizumab within 6 months from the first controlled status and 42% discontinue cyclosporine after 6 months regardless of the control status.

**Conclusions:**

The findings from the SDown‐CSU study clearly highlight a global need for guidance on the process of stepping down treatment in CSU. Additionally, the study offers a step‐down algorithm applicable to all stages of CSU treatment.

## 1 INTRODUCTION

Chronic spontaneous urticaria (CSU) is a common, mast cell‐driven, inflammatory skin disorder that affects more than 1% of the world's population.^1^, ^2^ CSU has a substantial burden on patients and society at large, markedly impairs patients' quality of life, and can be severely debilitating.^3^

The most important aim of CSU treatment is to achieve complete disease control based on the information obtained by the use of patient reported outcome measures (PROMs), primarily the urticaria control test (UCT) by achieving a UCT of 16, and a total improvement in quality of life (shown by CU‐QoL or DLQI) with the safest and most effective therapy.^4^ The utilization of PROMs has been facilitated by the launch of CRUSE, the UCARE chronic urticaria self‐evaluation app, with international and national versions available for download.^5^, ^6^ The most recent update of the International Urticaria Guideline recommends treating urticaria “until it is gone” and to do so “with as much as necessary and as little medication as possible” while encouraging clinicians to step up or step down the treatment of CSU based on levels of disease control assessed by the UCT.^1^

CSU is a self‐limiting disorder, with high rates of spontaneous remission within 2–5 years.^7^ Therefore, trials showing outcomes of stepping down strategies are needed, in patients with completely controlled CSU, to evaluate whether the disease is in spontaneous remission or controlled by the ongoing therapy. Currently, there is no established biomarker to discriminate between the two.^8^, ^9^ Apart from the need to check for spontaneous remission of the disease, other issues such as safety concerns, treatment burden, economic factors, pregnancy, onset of another disease, or intake of a new treatment may necessitate stepping down the treatment of CSU.^10^

Over the past decades, there have been significant advancements in the treatment of urticaria.^11^ As our knowledge of the pathogenesis of the disease increases, new targeted treatment options are being developed, and the near future of CSU treatment is promising. Nevertheless, once complete disease control is achieved with any therapy, the need for treatment continuation should always be assessed.

There remains a lack of clear guidance on when and how to step down the treatment in CSU patients, and expert consensus on step‐down protocols for first‐line, second‐line, and third‐line therapies is needed.^9^, ^10^, ^12^ The global network of Urticaria Centers of Reference and Excellence (UCAREs), specialized referral centers for the diagnosis and management of urticaria, aims to provide excellent patient care and CU management, increase the knowledge of urticaria, and promote the awareness of urticaria.^13^ The vast experience and expertise of urticaria experts at UCAREs may help with the development of guidance for stepping down strategies in CSU. Therefore, stepping down treatment in the CSU (SDown‐CSU) study assessed when, why, and how physicians at UCAREs around the world manage the stepping down of CSU treatment in their clinical practice. Based on this information, we developed guidance on the step down treatment in CSU and defined unmet needs in the field.

## 2 MATERIALS AND METHODS

### 2.1 Study design and participants

SDown‐CSU is an international, multicenter, observational, cross‐sectional, survey‐based study that was conducted as a UCARE Project. Every member of the UCARE network received an invitation to take part in the study, and all physicians who responded to the survey became study participants. Ethics approval was obtained from the coordinating center, Health Sciences University, Kayseri City Hospital (Decision date: 21.04.2022 and number: 616).

### 2.2 The SDown‐CSU questionnaire

The SDown‐CSU project steering committee members designed the aims, scope and plan of the project, and developed the questionnaire. After pilot testing of the questionnaire by the steering committee members, the final English version was developed and shared with the participating UCAREs. The investigated core variables were (1) the local regulations about stepping down/discontinuation of CSU treatments, (2) physicians' attitudes on different step down/discontinuation approaches, (3) physicians' attitudes on combined treatments, and (4) assessment of when and how to step down from different steps of CSU treatment. The questionnaire included 48 questions grouped into 4 main sections: (1) 19 questions about general information (2) 9 questions about omalizumab management, (3) 5 questions about cyclosporine management, and (4) 15 questions about treatment discontinuation (Suppl. Table S1).

### 2.3 PROM to assess disease control

In the SDown‐CSU questionnaire, questions about disease control were asked on the basis of UCT cut‐offs. UCT = 16 defines completely controlled CSU, 16 > UCT≥12 defines well‐controlled CSU, and UCT<12 defines uncontrolled CSU.

### 2.4 Data analysis

Due to the descriptive nature of the study, only the distribution of the responses to questions were presented and no statistical comparisons were performed.

## 3 RESULTS

### 3.1 General features of participating UCAREs

**FIGURE 1:**
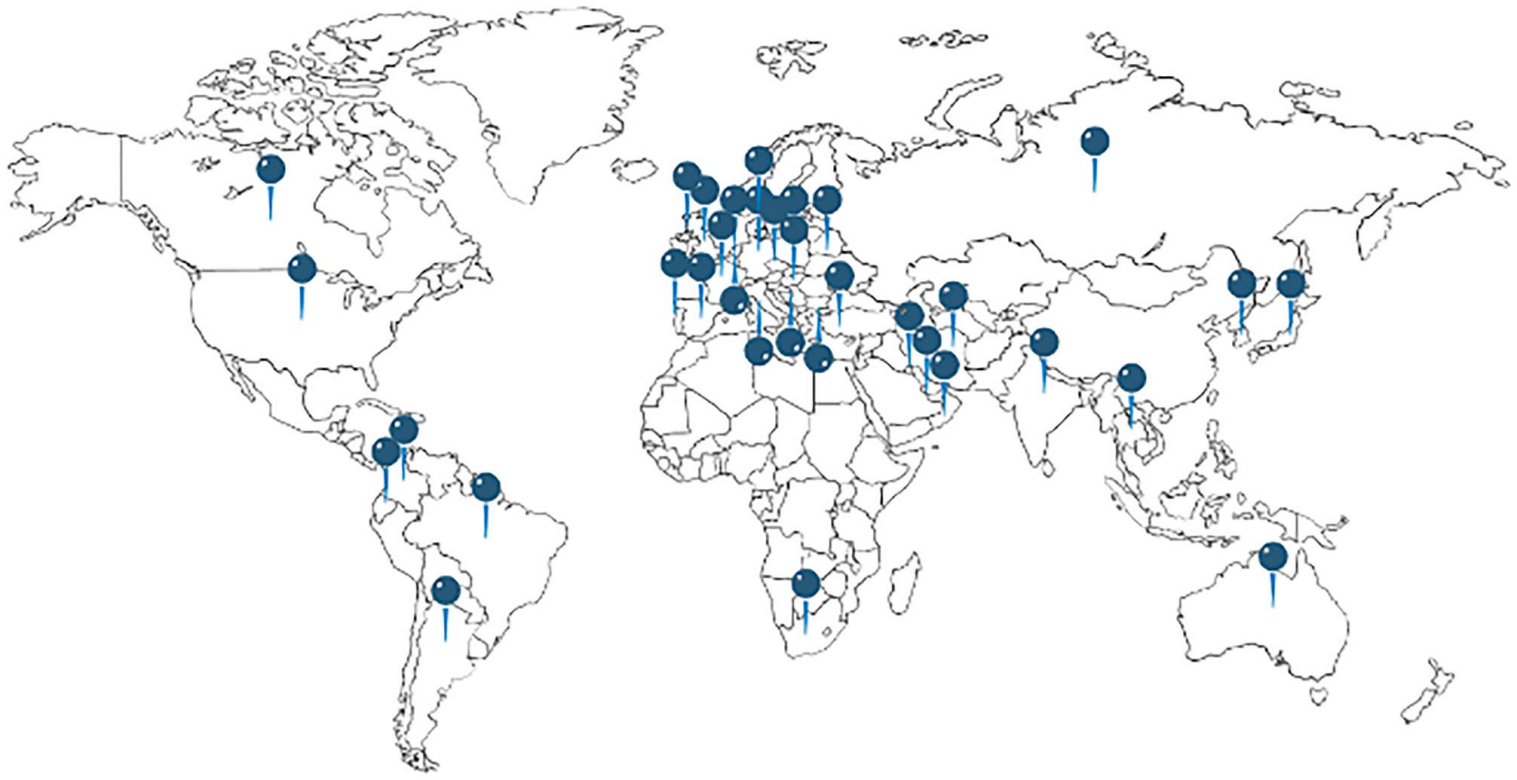
The global distribution of participating UCAREs of the SDown‐CSU study. One hundred and three physicians from 81 UCAREs in 34 different countries were involved.

Of 103 physicians from 81 UCAREs (Suppl. Table S2) from 34 countries and 6 continents (Figure 1), 59% were allergologists and 41% were dermatologists. Nearly all (97%) participants treated adult patients and 52%‐treated children with CSU. Forty and 17.5% took care of >50 and >100 CU patients per month, respectively. UCAREs with high numbers of patients per month were mostly from Turkey, South Korea and Italy. All of them treated adult patients.

### 3.2 National urticaria guidelines provide heterogeneous and insufficient guidance on stepping down CSU treatment

**FIGURE 2:**
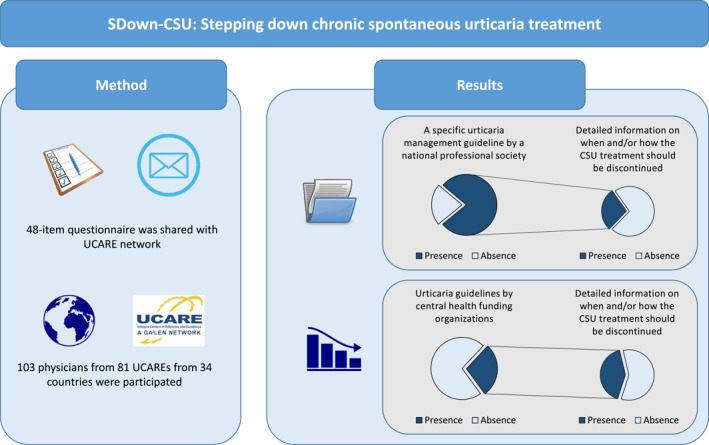
Urticaria guidelines by the central health funding organizations are rare and national recommendations mostly do not contain any detailed information on when and/or how CSU treatment should be discontinued.

A specific urticaria management guideline by a national professional society was reported by 78% of participants, with no national guidelines in Spain, South Africa, Slovenia, Oman, Qatar, Kuwait, Italy, Iran, Ireland and Ecuador. Three out of four (72%) guidelines did not provide detailed information on when and how CSU treatment should be discontinued once disease control is achieved (Table 1). Specifically, 82%, 78%, and 86% of the guidelines did not provide detailed guidance on when and how to step down antihistamines, omalizumab, and cyclosporine, respectively (Figure 2).

**TABLE 1:** Current status of the national guidelines proposed by national professional societies and central health funding organizations.

Information about national urticaria management guideline proposed by national professional societies	Information about regulatory guideline/paper instructing how to treat CSU, proposed by central health funding organizations
78% of the participants responded that they have a national urticaria management guideline proposed by national professional societies in their country	28% of the participants responded that they have regulatory guideline/paper instructing how to treat CSU, proposed by central health funding organizations in their country
72% of these guidelines do not contain any detailed information on when and/or how the CSU treatment should be discontinued	58.7% of these guidelines do not contain any detailed information on when and/or how the CSU treatment should be discontinued
82% of these guidelines do not contain detailed information on when and/or how the high dose AH treatment should be discontinued	83% of these guidelines do not contain detailed information on when and/or how the high dose AH treatment should be discontinued
78% of these guidelines do not contain detailed information on when and/or how omalizumab treatment should be discontinued	52% of these guidelines do not contain detailed information on when and/or how omalizumab treatment should be discontinued
86% of these guidelines do not contain detailed information on when and/or how cyclosporine treatment should be discontinued	86% of these guidelines do not contain detailed information on when and/or how cyclosporine treatment should be discontinued

### 3.3 Urticaria guidelines by health ministries or social security institutions are rare, and most do not provide guidance on stepping down CU treatment

Only 28.2% of the participants reported having a regulatory urticaria guideline issued by the ministry of health and/or social security institutions (Figure 2). All participants from Russia, Denmark, Italy, North Macedonia and Georgia responded “yes” to this question. Based on the responses of participants who reported having a regulatory guideline, ∼60% of these guidelines do not contain detailed information on when and how CSU treatment should be discontinued once disease control is achieved. Guidance on stepping down antihistamine treatment, omalizumab, and cyclosporine was missing from 83%, 52%, and 86% of regulatory guidelines, respectively (Table 1).

### 3.4 How UCAREs manage omalizumab and cyclosporine treatment

All physicians (99%) assessed patients for their response to omalizumab after at least 6 months of treatment, and three of four (76%) reported doing so within the first 3 months.

In patients with completely controlled CSU (UCT = 16), more than 90% of the physicians reported to make stepping down decisions within 6 months, after patients first achieved complete and sustained control. In patients with well‐controlled CSU (16 > UCT≥12), 90% of physicians reported that achieving well controlled but not completely controlled disease has them continue treatment with omalizumab, and only 5% reported to step down at this time (4% did not respond to this question). In omalizumab‐treated patients with uncontrolled (UCT<12) disease, 46% and 39% of physicians change the therapy within 3 and 6 months, respectively. Across all physicians, 70% reported that they increased omalizumab dosing to higher than 300 mg/4 weeks and 20% reported that local regulations do not allow to increase the dose. Of these who increased the omalizumab dose, 78% and 18%, respectively, reported to prefer 600 and 1200 mg every 4 weeks as the highest dose.

Physicians from 4 of 34 countries (South Africa, Iran, Canada and United Kingdom) reported that the initial duration of omalizumab treatment is limited to 6 months of maximum duration in their country (it is stated by the physicians that this limit is only to asses disease control and in case of treatment response or relapse after discontinuation, they have renewal criteria) (Table 2).

**TABLE 2:** Responses from UCAREs to questions about omalizumab and cyclosporine treatment.

Responses to questions regarding omalizumab treatment	Responses to questions regarding cyclosporine treatment
81% of the UCAREs responded that the duration of omalizumab is not limited with a predefined maximal duration in their country	82% of the UCAREs responded that the duration of cyclosporine is not limited with a predefined maximal duration in their country
76% assess control status of the patients under omalizumab treatment at least after 3 months and 99% do so within 6 months	92% of the UCAREs assess the control status of patients under cyclosporine treatment within 3 months
For patients with complete control (UCT = 16), 95% of the participants make stepping down decisions within 6 months from the first controlled status	Independent from the control status of the patient, 42% of the participants tend to discontinue cyclosporine within 6 months
For patients with well‐controlled (16 > UCT ≥12) disease, 90% of patients continue to treat and do no step down the treatment	32% of the UCAREs defined that they have no strict criteria about when to discontinue cyclosporine treatment and treat patients as long as needed
For patients with uncontrolled (UCT<12) disease, 46% and 39% of the UCARE physicians step‐up at least after 3 or 6 months from the first uncontrolled time interval, respectively	
Only 70% of the UCARE physicians increase omalizumab higher than the standard dose and they mostly prefer increasing omalizumab dose gradually starting from 450 mg to the maximum dose allowed in their country	
78% of the UCARE physicians that have access to higher than the standard doses of omalizumab, use a maximum dose of 600 mg/4 weeks	

Patients who are treated with cyclosporine were assessed for their levels of disease control by 53% and 92% of physicians within 1 and 3 months, respectively. 82% of the UCAREs responded that the duration of cyclosporine is not limited with a predefined maximal duration in their country.

### 3.5 How UCAREs step down CSU treatment

**FIGURE 3:**
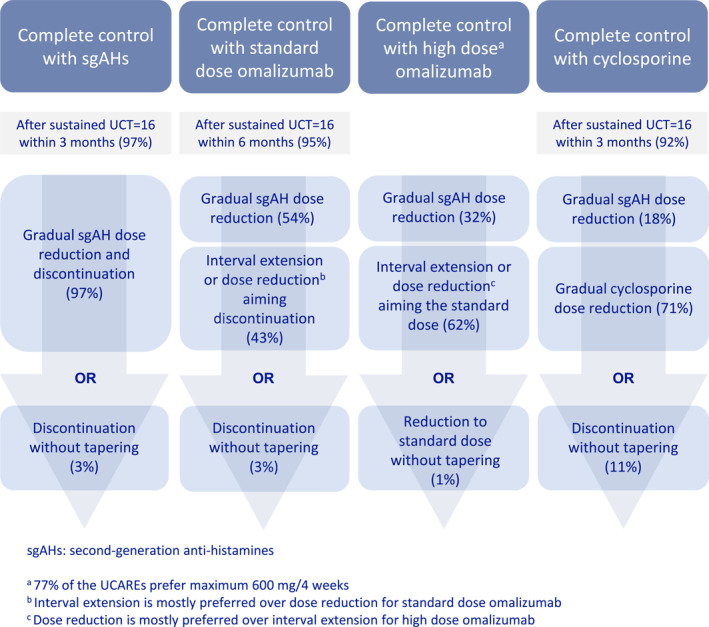
Preferences of the UCAREs for different steps of CSU treatment.

In patients with completely controlled CSU (UCT = 16) on high dose antihistamine treatment, 90% of physicians initiated step down within 3 months, and 97% of them stepped down gradually rather than abruptly stopped (Figure 3).

In patients with completely controlled CSU on standard‐dosed omalizumab and high dose antihistamine treatment, almost all physicians (95%) initiated omalizumab step down within the first 6 months of sustained complete control (median 3 [IQR: 1–3] months). Most physicians (54%) stepped down the antihistamine, whereas others (43%) preferred to step down omalizumab. When stepping down standard‐dosed omalizumab, 24% preferred to lower the omalizumab dose and 67% preferred to increase the treatment interval first. The remaining physicians, 9%, either prefer to discontinue omalizumab abruptly (8%) or do not have access to omalizumab in their country (1%). In contrast, in patients with a UCT of 16 on high dose omalizumab and high dose antihistamine treatment, most physicians (62%) preferred to first step down the omalizumab dose, whereas 32% of physicians favored stepping down the antihistamine dose first (6% of physicians did not indicate their preference).

In patients with completely controlled CSU in response to treatment with cyclosporine, together with high dose antihistamine treatment, 42%, 23%, and 3% of physicians reported discontinuing cyclosporine after a maximum treatment duration of 6, 12, and 24 months, respectively, whereas one third of physicians (32%) reported that they do not have a strict limit and treat the patients as long as needed.

Most physicians (72%) preferred stepping down cyclosporine first rather than antihistamines. Similar to omalizumab, most physicians (89%) preferred gradual dose reduction when stepping down cyclosporine. In case of relapse after discontinuation of all therapies, 79% of participants responded that they restart antihistamines before omalizumab.

## 4 DISCUSSION

This study, with a worldwide participation, demonstrates a global need for advice on stepping down treatment in CSU, with insufficient guidance provided using current international and national guidelines. Our findings address this need and provide a step‐down algorithm for CSU treatment reflecting the experience of urticaria experts from UCAREs. Effective step‐down strategies can help to avoid failed withdrawal attempts, save on drug use, and optimize the treatment overall.

The International Urticaria Guideline^1^ provides a step‐up algorithm for achieving complete disease control, but not a step‐down algorithm. In fact, our results show that there is very little guidance and advice on how to step down the treatment of CSU patients once they achieve complete disease control. Not all physicians have national guidelines on urticaria management and less than a third have a regulatory national guideline. Where such national guidelines exist, they hardly ever provide guidance on when and how to step down recommended therapies, that is, treatment with antihistamines, omalizumab, and cyclosporine. This needs to change and we call on the international urticaria guideline expert panel to consider including guidance on stepping down the treatment in the next update and revision of the guideline.

Omalizumab is the first and currently the only approved monoclonal antibody for the treatment of CSU. In CSU, omalizumab shows good long‐term efficacy and safety.^14^, ^15^, ^16^, ^17^ More than 80% of participants indicated that there is no limitation for the duration of omalizumab treatment in their country, suggesting that step down decisions are mainly based on the assessment of disease control achieved by treatment. Almost all of the participants indicated that they assess control status of the patients within 6 months of omalizumab treatment and 95% make stepping down decisions within 6 months from the first controlled status in patients with completely controlled disease. This indicates that, once patients achieve complete control, physicians are proactive in reducing or discontinuing omalizumab therapy to minimize treatment burden and costs while maintaining long‐term symptom control.

Completely controlled CSU is defined by the absence of any signs and symptoms of the disease while the patient is under treatment,^18^ and previous reports recommend stepping down attempts only in patients with completely controlled disease (UCT = 16).^10^, ^19^, ^20^ In case of well‐controlled disease (UCT≥12), the international urticaria guideline recommends continuing and optimizing the treatment in these patients.^1^ However, one third of physicians (35%) reported that they step down omalizumab in patients who did not achieve complete control. The reasons for this are unclear and need to be investigated. Complete disease control strongly relates to better sleep and quality of life as well as less impact on daily activities and work.^21^, ^22^ Treatment with omalizumab should only be stepped down in patients with complete control to see if spontaneous remission has occurred.

Cyclosporine is the third line treatment option recommended for patients with severe urticaria refractory to AHs and omalizumab.^1^ It is effective at low to moderate doses (3–5 mg/kg) and results in rapid clinical response, mostly within several days.^23^ The potential risk of significant adverse events including hypertension and renal impairment requires careful monitoring and generally limits its use beyond a few months, although there are some reports of long‐term use.^23^, ^24^ It is therefore surprising that more than 80% of the national or regulatory guidelines do not contain any information on how and when cyclosporine should be stepped down. Compared to omalizumab, UCAREs assess control status of patients treated with cyclosporine earlier, mostly within 3 months, probably due to concerns about side effects or unfamiliarity with safe prescribing of this drug.

How to step down and discontinue different steps of CSU treatment? Gradual reduction or abrupt discontinuation? As of now, no head‐to‐head trials exist comparing these stepping down approaches. Decisions should be based on individual factors, patient needs and local regulations. In patients with a complete response to high dose antihistamine therapy for 3 months, nearly all UCAREs prefer a gradual AH dose reduction. Previous expert reports support this and recommend reducing the daily dose by one tablet every month, with regular monitoring of signs and symptoms.^9^, ^10^ Omalizumab is an add‐on treatment to AHs and both treatments should be used together during stepping up. Although AHs are the mainstay of treatment of CSU, it was shown that patients with a complete response to omalizumab tend to discontinue their AH intake without losing complete control.^25^ Also, AH were shown to be effective for the treatment of intermittent symptoms in patients with well‐controlled urticaria under omalizumab treatment.^26^

When discontinuing omalizumab, UCAREs prefer interval prolongation over dose reduction for the standard dose, and dose reduction over interval prolongation for higher‐than standard doses. Interval prolongation is an effective off‐label recommendation, where retreating patients who show relapse^19^, ^27^ aid in finding the best individual injection interval.

When discontinuing cyclosporine, UCAREs prefer a gradual dose reduction rather than AH dose reduction. Interestingly, when stepping down standard‐dosed omalizumab and high dose antihistamine treatment, most (54%) of the physicians preferentially decreased the AH dose first, while when stepping down cyclosporine and high dose antihistamine treatment, only 17% preferred to decrease the AH dose. This may be due to efforts to shorten cyclosporine exposure as much as possible and to discontinue cyclosporine quickly due to concerns about its side effect profile.

If the disease relapses after treatment discontinuation, approximately 80% of UCARE physicians restart treatment with AHs first. A recent consensus by an expert panel recommended defining remission as the absence of CU signs and symptoms for 6 months while not on treatment.^18^ The reappearance of symptoms within less than 6 months should be regarded as relapse, whereas it is reasonable to define the onset of urticaria after more than 6 months, especially after 1 year, as a new urticaria episode. In case of disease relapse after discontinuation of the ongoing treatment, retreatment with the same drug is advised by previous studies.^9^, ^10^, ^11^, ^18^, ^19^

This study has some limitations. Firstly, it is a questionnaire‐based study. Although the results gained from the study are based on expert responses, evidence acquired from prospective studies is needed. Secondly, in some countries, social insurance regulations might differ between different states/regions and results may not reflect the regulations of the whole country. This study shows the experts' step down preferences based on their experience. We could suggest a gradual step down treatment in CSU with accurate surveillance of symptoms, but we cannot recommend the best protocol in order to prevent relapses and maintain the patients' well‐being based on the data available from this study.

Overall, the results of the SDown‐CSU study contribute to our understanding of the step‐down approaches for CSU treatment. The findings highlight the importance of individualized patient management, regular assessment of control status, and timely decision‐making for stepping down treatment based on disease control to avoid the burden and costs of prolonged treatment. The results of this study should help guide clinicians in the management of CSU and contribute to future updates of the International Urticaria Guidelines.

## AUTHOR CONTRIBUTIONS

**Murat Türk**: Conceptualization (lead); data curation (lead); formal analysis (lead); investigation (equal); methodology (equal); visualization (lead); writing ‐ original draft (lead); writing ‐ review & editing (equal). **Emek Kocatürk**: Conceptualization (lead); data curation (lead); formal analysis (equal); investigation (equal); methodology (equal); visualization (lead); writing ‐ original draft (equal); writing ‐ review & editing (equal); supervision (equal). **Ragıp Ertaş**: Conceptualization (equal); data curation (equal); investigation (equal); visualization (equal); writing ‐ review & editing (equal). **Luis Felipe Ensina**: Conceptualization (equal); data curation (equal); investigation (equal); visualization (equal); writing ‐ review & editing (equal). **Silvia Mariel Ferrucci**: Conceptualization (equal); data curation (equal); investigation (equal); visualization (equal); writing ‐ review & editing (equal). **Clive Grattan**: Conceptualization (equal); data curation (equal); investigation (equal); visualization (equal); writing ‐ review & editing (equal). **Christian Vestergaard**: Conceptualization (equal); data curation (equal); investigation (equal); visualization (equal); writing ‐ review & editing (equal). **Torsten Zuberbier**: Conceptualization (equal); data curation (equal); investigation (equal); visualization (equal); writing ‐ review & editing (equal). **Marcus Maurer**: Conceptualization (lead); data curation (lead); formal analysis (equal); investigation (equal); methodology (equal); visualization (lead); writing ‐ original draft (equal); writing ‐ review & editing (equal); supervision (equal). **Ana Maria Giménez‐Arnau**: Conceptualization (lead); data curation (lead); formal analysis (equal); investigation (equal); methodology (equal); visualization (lead); writing ‐ original draft (equal); writing ‐ review & editing (equal); supervision (equal).

## CONFLICT OF INTEREST STATEMENT

Murat Türk is or recently was a speaker and/or advisor for AstraZeneca, GSK, Novartis, Roxall, Vem İlaç. Emek Kocatürk is or recently was a speaker and advisor for Novartis, Menarini, LaRoche Posey, Sanofi, Bayer, Abdi İbrahim, Pfizer. Ragıp Ertaş is or recently was a speaker and/or advisor for Novartis, Abbvie, Jannsen, and Pfizer. Luis Felipe Ensina is or recently was a speaker and/or advisor for and/or has received research funding from Abbvie, Novartis and Sanofi. Silvia Mariel Ferrucci is or recently was a speaker and/or advisor for and/or has received research funding from ABBVIE, Almirall, Galderma, Leo Pharma, Sanof, Amgen, Novartis, Bayer, Novartis and Menarini. Clive Grattan has sat on Advisory Boards for Celltrion and Blueprint Medicines. He is on Steering Committees for Blueprint Medicines and AB Science. Christian Vestergaard is or recently was a speaker and advisor for Novartis, Sanfoi Genzyme, Ely Lilly, Pfizer, Almirall, LEO Pharma, Astra Zeneca, Pierre Fabre, OM Pharma, and Abbvie. Torsten Zuberbier has received industry consulting, research grants, and/or honoraria from AImmune, AjantaPharma, Astra Zeneca, AbbVie, ALK, Almirall, Astellas, Bayer Health Care, Bencard, Berlin Chemie, BioCryst, Celldex, FAES, HAL, Henkel, Kryolan, Leti, Lofarma, L’Oreal, Meda, MediWound, Menarini, Merck, MMV Medicines for Malaria Venture, MSD, Novartis, PCM Scientific, Pfizer, Sanofi, Sanoflore, Stallergenes, Takeda, Teva, and UCB; and has organizational affiliations with WHO‐Initiative “Allergic Rhinitis and its Impact on Asthma” (ARIA) (committee member), German Society for Allergy and Clinical Immunology (DGAKI) (member of the board), European Centre for Allergy Research Foundation (ECARF) (chairman of the board), Global Allergy and Asthma European Network (GA2LEN) (president), and Committee on Allergy Diagnosis and Molecular Allergology, World Allergy Organisation (WAO) (member). Marcus Maurer is or recently was a speaker and/or advisor for and/or has received research funding from Allakos, Alvotech, Amgen, Aquestive, Aralez, AstraZeneca, Bayer, Celldex, Celltrion, Evommune, GSK, Ipsen, Kyowa Kirin, Leo Pharma, Lilly, Menarini, Mitsubishi Tanabe Pharma, Moxie, Noucor, Novartis, Orion Biotechnology, Resoncance Medicine, Sanofi/Regeneron, Septerna, Trial Form Support International AB, Third HarmonicBio, ValenzaBio, Yuhan Corporation, Zurabio. Ana Maria Giménez‐Arnau is or recently was a speaker and/or advisor for and/or has received research funding from Almirall, Amgen, AstraZeneca, Avene, Celldex, Escient Pharmaceuticals, Genentech, GSK, Instituto Carlos III‐ FEDER, Leo Pharma, Menarini, Novartis, Sanofi–Regeneron, Servier, Thermo Fisher Scientific, Uriach Pharma/Neucor.

## Supporting information

Supporting Information S1

## Data Availability

Raw data were generated at Kayseri City Hospital. Derived data supporting the findings of this study are available from the corresponding author on request.
