# Supplementary material for: A global perspective on stepping down chronic spontaneous urticaria treatment: Results of the Urticaria Centers of Reference and Excellence SDown‐CSU study
Source: Clin Transl Allergy. 2024 Feb 14;14(2):e12343. doi: 10.1002/clt2.12343 (PMC10865765; doi:10.1002/clt2.12343)
Supplement: Supplementary file 1 — Supporting Information S1 [file CLT2-14-e12343-s001.docx]

**Supplementary Table 1.** The SDown-CSU questionnaire.

| 1. **General Information** |
| --- |
| 1. Physician’s name …………………... |
| 1. Center’s name …………………... |
| 1. Country of your center …………………... |
| 1. City of your center …………………... |
| 1. Your specialty: 2. Dermatology 3. Pediatric Allergy-immunology 4. Adult Allergy-immunology 5. Other; please specify …………………... |
| 1. Your specialty: 2. Dermatology 3. Pediatric Allergy-immunology 4. Adult Allergy-immunology 5. Other; please specify …………………... |
| 1. Which age group of CSU patients are you treating? 2. Adult 3. Pediatrics 4. Both |
| 1. Approximate number of chronic urticaria patients (including first diagnoses and follow-up patients) followed up per month:    1. 0-10    2. 11-25    3. 26-50    4. 51-100    5. >100 |
| 1. Is there any national urticaria management guideline proposed by national professional societies in your country? 2. Yes 3. No |
| 1. If yes to Q#8, does this guideline involve detailed information on when and/or how the CSU treatment should be discontinued once the disease control is maintained? (You can select more than one answer) 2. Yes, this guideline involves detailed information on when the CSU treatment should be discontinued once the disease control is maintained 3. Yes, this guideline involves detailed information on how the CSU treatment should be discontinued once the disease control is maintained 4. No 5. Other …………………... |
| 1. If yes to Q#8, does this guideline involve detailed information on when and/or how the high dose antihistamine (AH) treatment should be discontinued in CSU once the disease control is maintained? (You can select more than one answer) 2. Yes, this guideline involves detailed information on when the high dose antihistamine treatment should be discontinued 3. Yes, this guideline involves detailed information on how the high dose antihistamine treatment should be discontinued 4. No 5. Other …………………... |
| 1. If yes to Q#8, does this guideline involve detailed information on when and/or how the omalizumab treatment should be discontinued in CSU once the disease control is maintained? (You can select more than one answer) 2. Yes, this guideline involves detailed information on when the omalizumab treatment should be discontinued 3. Yes, this guideline involves detailed information on how the omalizumab treatment should be discontinued 4. No 5. Other …………………... |
| 1. If yes to Q#8, does this guideline involve detailed information on when and/or how the cyclosporine treatment should be discontinued in CSU once the disease control is maintained? (You can select more than one answer) 2. Yes, this guideline involves detailed information on when the cyclosporine treatment should be discontinued 3. Yes, this guideline involves detailed information on how the cyclosporine treatment should be discontinued 4. No 5. Other …………………... |
| 1. Is there any regulatory guideline/paper instructing how to treat CSU, proposed by ministry of health and/or social security institutions, in your country? 2. Yes 3. No |
| 1. If yes to Q#13, does the guideline/paper involve detailed information on when and how the CSU treatment should be discontinued once the disease control is maintained? 2. Yes, the guideline/paper involve detailed information on when and how the CSU treatment should be discontinued once the disease control is maintained 3. Yes, the guideline/paper involve detailed information only on when the CSU treatment should be discontinued once the disease control is maintained 4. Yes, the guideline/paper involve detailed information only on how the CSU treatment should be discontinued once the disease control is maintained 5. No 6. Other …………………... |
| 1. If yes to Q#13, does the guideline/paper involve detailed information on when and how the AH treatment should be discontinued once the disease control is maintained? 2. Yes 3. No 4. Other …………………... |
| 1. If yes to Q#13, does the guideline involve detailed information on when and how the omalizumab treatment should be discontinued once the disease control is maintained? 2. Yes 3. No 4. Other …………………... |
| 1. If yes to Q#13, does the guideline involve detailed information on when and how the cyclosporine treatment should be discontinued once the disease control is maintained? 2. Yes 3. No 4. Other …………………... |
| 1. Do you think EAACI/GA^2^LEN/EDF/WHO chronic urticaria guideline has adequate content on how the treatment should be discontinued once the disease control is maintained in CSU? 2. Yes 3. No 4. Other …………………... |
| 1. **Questions about omalizumab management** |
| 1. Independent from the control status of the patient, is the duration of ongoing omalizumab treatment limited with a predefined maximal duration in your country? 2. Yes 3. No |
| 1. When do you assess the control status (to define controlled or uncontrolled) of the patient treated with omalizumab for the first time? 2. At least after 1 month 3. At least after 3 months 4. At least after 6 months 5. At least after 12 months 6. Other …………………... |
| 1. How do you manage antihistamine therapy in a patient started on omalizumab treatment? 2. Resume high dose antihistamines from the beginning to the end of omalizumab therapy 3. Gradual AH dose reduction according to control status of the patient 4. Abrupt AH discontinuation after controlled disease 5. Abrupt AH discontinuation with start of omalizumab treatment 6. Other …………………... |
| 1. For how long do you treat a completely controlled (UCT=16) patient with sustained omalizumab therapy before deciding to step down/discontinuation? (You can select more than one)    1. I start step down immediately after complete control    2. At least 1 month from the first controlled status    3. At least 3 months from the first controlled status    4. At least 3 months from the first dose    5. At least 6 months from the first controlled status    6. At least 6 months from the first dose    7. At least 12 months from the first controlled status    8. At least 12 months from the first dose    9. Other …………………... |
| 1. For how long do you treat a well-controlled (16>UCT≥12) patient with sustained omalizumab therapy before deciding to step down/discontinuation? (You can select more than one)    1. I start step down immediately after well-controlled disease    2. At least 3 months from the first controlled status    3. At least 3 months from the first dose    4. At least 6 months from the first controlled status    5. At least 6 months from the first dose    6. At least 12 months from the first controlled status    7. At least 12 months from the first dose    8. I don’t step down/discontinue    9. Other …………………... |
| 1. For how long do you treat an uncontrolled (UCT<12) patient with sustained omalizumab therapy before deciding to change the therapy?    1. I change immediately after I realize it is not under control    2. At least 3 months from the first uncontrolled time interval    3. At least 6 months from the first uncontrolled time interval    4. At least 12 months from the first uncontrolled time interval    5. Other …………………... |
| 1. In a patient with uncontrolled disease under standard dose of omalizumab, do you increase the dose of omalizumab (higher than 300 mg / 4 weeks)?    1. Yes, I do    2. No, I do not, because it is not possible in my country due to regulations    3. No, I do not, I do not find it effective    4. No, I do not, other reasons |
| 1. If you increase the dose of omalizumab, what is the maximum omalizumab dose you prefer in your clinical practice?    1. 450 mg/4 weeks    2. 600 mg/4 weeks    3. 750 mg/4 weeks    4. 900 mg/4 weeks    5. 1050 mg/4 weeks    6. 1200 mg/4 weeks |
| 1. When there is incomplete control or no control after 6 doses of omalizumab 300 mg/4 weeks, which one is your preference?    1. Increase omalizumab dose gradually starting from 450 mg to the maximum dose    2. Increase the dose directly to the maximum dose    3. Add cyclosporine to omalizumab    4. Add corticosteroids to omalizumab    5. Quit omalizumab and switch to cyclosporine    6. Others, please specify…………………………………… |
| 1. **Questions about cyclosporine management** |
| 1. Independent from the control status of the patient, is the duration of ongoing cyclosporine treatment limited with a predefined maximal duration in your country? 2. Yes 3. No |
| 1. When do you assess the control status (to define controlled or uncontrolled) of the patient treated with cyclosporine for the first time? 2. After 1 month 3. At least after 3 months 4. At least after 6 months 5. At least after 12 months 6. Other …………………... |
| 1. How do you manage antihistamine therapy in a patient started on cyclosporine treatment? 2. Resume high dose antihistamines from the beginning to the end cyclosporine therapy 3. Gradual AH dose reduction according to control status of the patient 4. Abrupt AH discontinuation after controlled disease 5. Abrupt AH discontinuation after starting cyclosporine treatment 6. Do not prefer AHs with cyclosporine 7. Other …………………... |
| 1. Independent from the control status of the patient, for how long do you continuously treat a patient with cyclosporine? 2. Max. 3 months 3. Max. 6 months 4. Max. 12 months 5. I do not have a limit; I treat as needed 6. Other …………………... |
| 1. Independent from the control status of the patient, for how long do you maximally (combined cumulative dose of all cyclosporine treatments from different time points) treat a patient with cyclosporine? 2. Max. 3 months 3. Max. 6 months 4. Max. 12 months 5. Max. 24 months 6. No strict maximal cumulative dose 7. Other …………………... |
| 1. **Questions about treatment discontinuation** |
| 1. In your clinical practice, when do you start step down approaches in a patient with controlled disease under high dose anti-histamine therapy? 2. At least after 1 month with controlled disease 3. At least after 3 months with controlled disease 4. At least after 6 months with controlled disease 5. Other …………………... |
| 1. Independent from the local regulations of your country, which step down approach below is the most suitable for a patient with complete controlled CSU under high dose AH therapy?    1. If I am convinced that the patient was treated for a sufficient time, I prefer to step down gradually the AH dose first    2. If I am convinced that the patient was treated for a sufficient time, I discontinue the AH abruptly    3. Other …………………... |
| 1. According to your local regulations, which step down approach below is the most suitable for a patient with complete controlled CSU under high dose AH therapy for at least 3 months?    1. No significant change for local regulations, same approach with Q#34    2. If the patient was treated for a sufficient time, step down the AH dose gradually first, then discontinuation    3. If the patient was treated for a sufficient time, discontinuation of the AHs abruptly    4. Other …………………... |
| 1. Independent from the local regulations of your country, which step down approach below is the most suitable for a patient with completely controlled CSU under high dose AH added-on standard dose omalizumab therapy (300 mg / 4 weeks) ?    1. If I am convinced that the patient was treated for a sufficient time, I prefer to decrease the AH dose first    2. If I am convinced that the patient was treated for a sufficient time, I prefer to decrease omalizumab dose or increase the treatment interval first    3. If I am convinced that the patient was treated for a sufficient time, I discontinue all treatments abruptly    4. Other …………………... |
| 1. According to your local regulations, which step down approach below is the most suitable for a patient with complete controlled CSU under high dose AH added-on standard dose omalizumab therapy (300 mg / 4 weeks) ?    1. No significant change for local regulations, same approach with Q#34    2. If the patient was treated for a sufficient time, decreasing the AH dose first    3. If the patient was treated for a sufficient time, decreasing omalizumab dose or increasing the treatment interval first    4. If the patient was treated for a sufficient time, discontinuation of all treatments abruptly    5. Other …………………... |
| 1. In a patient with controlled disease under 300 mg / 4 weeks omalizumab therapy, which step down approaches you prefer in your daily clinical practice? (You can select more than one answer)    1. Dose reduction of omalizumab    2. Interval prolongation of omalizumab    3. Abrupt discontinuation    4. Other …………………... |
| 1. Independent from the local regulations of your country, which step down approach below is the most suitable for a patient with complete controlled CSU under high dose AH added-on cyclosporine?    1. If I am convinced that the patient was treated for a sufficient time, I prefer to decrease the AH dose first    2. If I am convinced that the patient was treated for a sufficient time, I prefer to decrease cyclosporine dose first, without changing the AH dose    3. If I am convinced that the patient was treated for a sufficient time, I discontinue all treatments abruptly    4. I do not use AH treatment with cyclosporine treatment    5. Other …………………... |
| 1. According to your local regulations, which step down approach below is the most suitable for a patient with complete controlled CSU under high dose AH added-on cyclosporine?    1. No significant change for local regulations, same approach with Q#39    2. If the patient was treated for a sufficient time, decreasing the AH dose first    3. If the patient was treated for a sufficient time, decreasing cyclosporine dose first without changing the AH dose    4. If the patient was treated for a sufficient time, discontinuation of all treatments abruptly    5. Other …………………... |
| 1. In a patient with controlled disease under cyclosporine therapy, which step down approach you select first in your daily clinical practice?    1. Dose reduction, then discontinuation of cyclosporine    2. Abrupt discontinuation of cyclosporine    3. Other …………………... |
| 1. Independent from the local regulations of your country, which step down approach below is the most suitable for a patient with completely controlled CSU under high dose AH added-on HIGH DOSE omalizumab therapy (max 600 mg / 4 weeks)?    1. If I am convinced that the patient was treated for a sufficient time, I prefer to decrease the AH dose first    2. If I am convinced that the patient was treated for a sufficient time, I prefer to decrease omalizumab dose first    3. If I am convinced that the patient was treated for a sufficient time, I discontinue all treatments abruptly    4. Other …………………... |
| 1. According to your local regulations, which step down approach below is the most suitable for a patient with completely controlled CSU under high dose AH added-on HIGH DOSE omalizumab therapy (max 600 mg / 4 weeks) ?    1. No significant change for local regulations, same approach with Q#42    2. If the patient was treated for a sufficient time, decreasing the AH dose first    3. If the patient was treated for a sufficient time, decreasing omalizumab dose first    4. If the patient was treated for a sufficient time, discontinuation of all treatments abruptly    5. Other …………………... |
| 1. In a patient with completely controlled CSU for at least 3 months under AHs and standard dose of omalizumab therapy, do you think decreasing the dose and/or complete discontinuation of AHs without changing omalizumab therapy is an appropriate method?    1. Yes, I use this approach in my daily clinical practice    2. Yes, but I cannot use it due to local regulations in my country    3. Yes, but I cannot use it due to low level of evidence    4. My approach is based on individual patient; I use them adjusted according to patients’ need    5. No, I believe patient should at least use daily 1x1 AH    6. Other …………………... |
| 1. In a patient with completely controlled CSU for at least 3 months under AHs and cyclosporine therapy, do you think decreasing the dose and/or complete discontinuation of AHs without changing cyclosporine therapy is an appropriate method?    1. Yes, I use this approach in my daily clinical practice    2. Yes, but I cannot use it due to local regulations in my country    3. Yes, but I cannot use it due to low level of evidence    4. My approach is based on individual patient; I use them adjusted according to patients’ need    5. No, I believe patient should at least use daily 1x1 AH    6. Other …………………... |
| 1. In a patient with completely controlled CSU under omalizumab therapy, do you think using AHs as needed is an appropriate method to suppress intermittent symptoms occurred during the interval?    1. Yes, I use this approach in my daily clinical practice    2. Yes, but I cannot use it due to local regulations in my country    3. Yes, but I cannot use it due to low level of evidence    4. No, I believe patient should at least use daily 1x1 AH    5. Other …………………... |
| 1. After discontinuation of all therapies (omalizumab + antihistamines) in a patient with complete controlled disease, if the disease relapses, which retreatment option do you prefer first? 2. Starting standard dose omalizumab first 3. Starting antihistamines first 4. Starting antihistamines and omalizumab together 5. Other …………………… |

**Supplementary table 2.** Participants Urticaria Centers of Reference and Excellences and their countries.

| **Countries** | **Participant UCAREs** |
| --- | --- |
| Argentina | Fundacion Ayre |
|  | Hospital Italiano de Buenos Aires |
|  | IAAER |
|  | Instituto de Alergia e Inmunologia del Sur (IAIS) |
| Australia | Box Hill Hospital, Melbourne |
| Austria | Kepler Uniklinikum |
| Brazil | Alergoalpha/CPAlpha |
|  | Alergoskin Alergia e Dermatologia SS LTDA |
|  | Clementino Fraga Filho University Hospital |
|  | FEDERAL UNIVERSITY OF PARANÁ |
|  | State University of Rio de Janeiro |
|  | UCARE - JUIZ DE FORA |
|  | UCARE FACULDADE DE MEDICINA DO ABC (ABC MEDICAL SCHOOOL) |
|  | Uiversity of Campinas (UNICAMP) |
|  | Universidade Federal da Bahia: C-HUPES |
| Bulgaria | Clinic of Allergology, University Hospital Alexandrovska, Medical University of Sofia |
|  | Medical Centre Excelsior |
| Canada | Clinique Lacroix |
|  | Montreal Children's Hospital |
| Colombia | GROUP OF CLINICAL AND EXPERIMENTAL ALLERGY |
|  | Universidad de Antioquia |
| Denmark | Aarhus University hospital |
|  | Bispebjerg Hospital |
| Ecuador | Respiralab |
| France | Grenoble |
|  | Tenon-APHP-Paris UCARE center |
| Georgia | Center of Allergy and Immunology |
| Germany | Charité - Universitätsmedizin Berlin |
|  | Department of Dermatology University Medical Center Mainz |
|  | Department of Dermatology, University Hospital Essen |
|  | Erlangen |
|  | Klinik für Dermatologie Elbe Klinikum Buxtehude |
|  | University Hospital Carl Gustav Carus Dresden |
| India | College of Medicine and Sagore Dutta Hospital, Kolkata, Indial |
|  | DR D Y PATIL MEDICAL COLLEGE AND HOSPITAL |
| Iran | Allergy Research Center, Department of Allergy and Immunology, Mashhad University Of Medical Sciences |
| Ireland | St. James's Hospital |
| Italy | Ambulatorio di Allergologia, Clinica San Carlo, Paderno Dugnano (MI) |
|  | Fondazione IRCCS Ca' Granda Ospedale Maggiore Policlinico di Milan |
| Japan | Hiroshima Univeristy, Department of Dermatology |
|  | Kobe University, Osaka Medical and Pharmaceutical University |
| Kuwait | Kuwait allergy center |
| North Macedonia | Remedika general hospital |
| Oman | Royal Hospital |
| Poland | Barlicki Memorial Hospital UCARE |
|  | Department of Dermatology, University of Rzeszow |
|  | Department of Internal Diseases and Allergology |
|  | European Center for Diagnosis and Treatment of Urticaria/Angioedema (GA2LEN UCARE /ACARE Network) & Department of Clinical Allergology and Urticaria of Medical University of Silesia, Poland 41-800 Zabrze ul. Marii Curie-Skłodowskiej 10 |
|  | Kasperska-Zajac |
|  | UCAREPOLAND, LODZ CSK - CENTRAL UNIVERSITY HOSPITAL |
| Portugal | Dermatology - Coimbra University Hospital |
|  | Immunoalergology Department - Centro Hospitalar Vila Nova de Gaia / Espinho |
|  | Patologia cutânea, Centro Hospitalar Vila Nova de Gaia |
| Qatar | Hamad medical corporation |
|  | Qatar center of excellence allergy and immunology |
| Russia | NRC Institute of Immunology |
|  | First Moscow State Medical University |
|  | Smolensk State Medical University |
| Slovenia | University Clinic of Respiratory and Allergic Diseases Golnik |
| South Africa | University of Cape Town |
| South Korea | Ajou University |
|  | Dong-A University hopital |
|  | Hallym University Sacred Heart Hospital |
|  | Keimyung University Dongsan Hospital |
|  | Yeungnam University Medical Center. |
| Spain | Hospital del Mar. IMIM. Universitat Pompeu Fabra |
|  | Vall d'Hebron Hospital. Allergy Service |
| Thailand | Siriraj Urticaria and Angioedema Center |
| Netherlands | Erasmus MC |
| Turkey | Başakşehir Çam and Sakura city Hospital |
|  | Bezmialem Vakif University Department of Dermatology |
|  | Erciyes University School of Medicine Department of Chest Diseases Division of Immunology and Allergic Diseases |
|  | Hacettepe University |
|  | Istanbul Faculty of Medicine, Adult Allergy and Immunology Clinic |
|  | Kayseri City Hospital |
|  | Kırşehir Ahi Evran Üniversitesi Tıp Fakültesi |
|  | Koç University |
|  | Sakarya University Training and Research Hospital, Department of Dermatology |
|  | Sivas Cumhuriyet University, School of Medicine, Dermatology Department |
| United Kingdom | London Allergy and Immunology Centre |
| USA | University of Cincinnati College of Medicine and Bernstein Allergy Group and Clinical research center |
| Wales | University Hospital of Wales |
